# DTI Study of Cerebral Normal-Appearing White Matter in Hereditary Neuropathy With Liability to Pressure Palsies (HNPP)

**DOI:** 10.1097/MD.0000000000001909

**Published:** 2015-10-30

**Authors:** Wei-wei Wang, Chun-li Song, Liang Huang, Qing-wei Song, Zhan-hua Liang, Qiang Wei, Jia-ni Hu, Yan-wei Miao, Bing Wu, Lizhi Xie

**Affiliations:** From the Radiology Department of the First Affiliated Hospital of Dalian Medical University, Dalian, Liaoning, PR China (WWW, SQW, WQ, MYW); Neurology Department of the First Affiliated Hospital of Dalian Medical University, Dalian, Liaoning, PR China (SCL, HL, LZH); Department of Radiology, Wayne State University, Detroit, Michigan, USA (HJN); and GE Healthcare China, Beijing, PR China (WB, XLZ).

## Abstract

The majority of previous studies on hereditary neuropathy with liability to pressure palsies (HNPP) were focused on peripheral nerves, whereas cerebral alterations in HNPP have been less attended to. In this work, Diffusion tensor imaging (DTI) was used to detect the changes in WM, especially in the normal-appearing white matter (NAWM) in HNPP patients for its sensitivity in probing the microstructure of WM, the sensitive metric was searched for probing cerebral alterations and the regional distribution of cerebral abnormalities was identified. Twelve HNPP patients and 12 age- and gender-matched healthy controls underwent the conventional MRI, DTI scan, and electrophysiological examination. The conventional MRI images were first analyzed to identify abnormal intense regions and the NAWM regions. NAWM refers to the white matter regions that do not include the lesions on conventional MRI. The apparent diffusion coefficient and fractional anisotropy (FA) values of the NAWM were then measured and compared between patient and control groups. The sensitivity and specificity of 3 methods and the cerebral regional distribution of MR signal abnormalities were further analyzed. Hyperintense foci were observed on T2 weighted image and fluid attenuated inversion recovery images in 6 patients. Compared to the controls, FA values of the patients were significantly lower in bilateral frontal, orbitofrontal, and temporal NAWMs; whereas the electrophysiological examination results of patients and controls exhibited no statistically significant difference. The sensitivity of FA value was higher than that of electrophysiological examination and conventional MRI. The majority of abnormal signals on conventional MRI images and abnormal FA values were located in the frontal and temporal lobes. The results of our study show cerebral WM changes in HNPP patients. FA value in DTI has been shown to be sensitive to the cerebral microstructural changes in HNPP. The frontal lobe is the predilection site that is most involved in HNPP.

## INTRODUCTION

Hereditary neuropathy with liability to pressure palsies (HNPP) is a rare autosomal-dominant inherited disease, which was initially described by De Jong in 1947.^1^ HNPP typically presents with recurrent painless entrapment neuropathies at vulnerable sites including peroneal, median, and ulnar nerves.^2^ The pathological alterations in peripheral nerve system can be explicitly observed on peripheral nerve biopsy,^3^ which shows the tomaculae resulting from recurrent demyelination and myelin reduplication.

The majority of the previous studies on HNPP were concentrated on peripheral nerves using electrophysiological examination.^4–8^ Several case reports have investigated cerebral involvements by using MRI, which showed hyperintense foci in cerebral WM.^9–12^ However, CNS symptoms, such as face numbness, hemiparesis, hypoesthesia, transient visual field defects, acute vocal cord and hypoglossal nerve paralysis,^9,10,13,14^ cannot be fully explained by the hyperintense foci on conventional MRI. To date, there is only 1 Diffusion tensor imaging (DTI) study demonstrating the decreased fractional anisotropy (FA) in WM lesions.^15^ However, the involvement of normal-appearing WM (NAWM) and the distribution of cerebral lesions in HNPP were not thoroughly described in this study. In the present study, it was hypothesized that the NAWM on the proximity to the observed lesions might be involved. NAWM refers to the white matter regions that do not include the lesions on conventional MRI. DTI is a recently developed imaging method to perform sensitive detection of WM disorders, especially those involving NAWM.^16–18^ Therefore, the purpose of this study was to detect WM changes, especially the NAWM in HNPP patients by utilizing DTI, search for a sensitive metric to probe cerebral alterations and identify the regional distribution of cerebral abnormalities.

## PATIENTS AND METHODS

### Patients and Controls

This study was approved by institutional review board of the First Affiliated Hospital of Dalian Medical University, and written informed consent was obtained from all the subjects after fully explanation of the nature of the procedures. Considering the concentration of the present study was on WM changes, the following inclusion criteria were developed for patient selection: no history of head injury, brain tumors, cerebrovascular disorder, hypertension, hyperlipemia, diabetes mellitus, thyroid disease, or alcohol/substance dependence. Three patients with hypertension or diabetes mellitus were excluded. Twelve patients (5 males and 7 females, age: 35.6 ± 13.6, ranging from 11 to 54 years old) diagnosed by a gene test that showed a consistent deletion of PMP22 gene with HNPP were included ultimately. The patients group was consisted of 5 unrelated pedigrees. The peripheral nerves symptoms included limbs numbness, weakness, and pain. The CNS symptoms included dizziness, impaired memory, sleep disorders, headache, impaired vision, and hyperhidrosis. The general physical examination of all patients indicated normal conditions. Tendon reflex of limbs was reduced or absent. Additionally, for all the patients, Babinski sign, Chaddock sign, Gonda sign, and Hoffmann sign were negative and mini-mental state examination (MMSE) scores remained within normal range (28.25 ± 1.29). Twelve age- and gender-matched healthy subjects (5 male, 7 female; mean age, 35.6 ± 14 years; age range, 11–55 years) were recruited as controls who underwent gene test to preclude the possibility of having HNPP. Exclusion criteria for the controls were as follows: alcohol/substance dependence; subjects with abnormal intensity on conventional MRI. Moreover, all the subjects were right-handed. All the subjects underwent MRI and electrophysiological examination without any medicine or alcohol intake within 48 hours before the examinations. The detailed demographic and clinical information of subjects was summarized in Table 1.

**TABLE 1:**
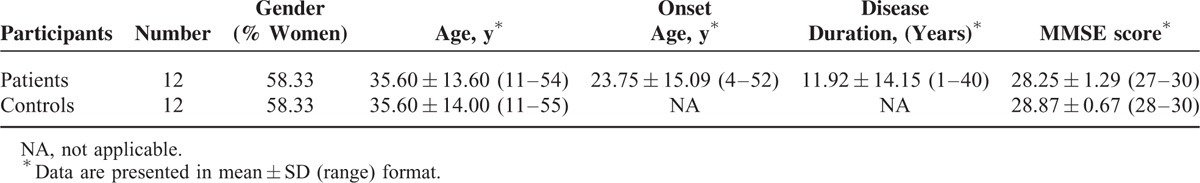
Demographic Features and Clinical Information of HNPP Patients and Controls

### MRI Acquisition Protocol

All patients and controls underwent scanning on 3-T MR system (GE Signa 3.0T HD echospeed MRI) using an eight channel phased-array brain coil. MRI images were obtained for both conventional MR [Sagittal T1WI, axial T1WI, axial T2 weighted image (T2WI), and axial T2 fluid attenuated inversion recovery (FLAIR)] and DTI sequence. DTI images were acquired by using single-shot spin echo planar imaging protocol. Twenty-five diffusion directions were performed using b-values of 0 and 1000 s/mm^2^, together with B_0_ correction. ASSET technology was used with an acceleration factor of 2. Acquisition time of DTI sequence was approximately 270 seconds. The detailed parameters of each sequence are shown as follow. Sag T1WI: slice thickness = 6 mm, slice gap = 1 mm, TR = 2500 ms, TE = 24 ms, FOV = 24 cm × 24 cm, Matrix = 320 × 224, NEX = 1, Phase FOV = 1. Ax T1WI: slice thickness = 6 mm, slice gap = 1 mm, TR = 2250 ms, TE = 24 ms, FOV = 24 cm × 24 cm, Matrix = 320 × 256, NEX = 1, Phase FOV = 0.9. Ax T2WI: slice thickness = 6 mm, slice gap = 1 mm, TR = 5000 ms, TE = mini, FOV = 24 cm × 24 cm, Matrix = 256 × 256, NEX = 2, Phase FOV = 0.8. Ax T2 FLAIR: slice thickness = 6 mm, slice gap = 1 mm, TR = 9000 ms, TE = 168 ms, FOV = 24 cm × 24 cm, Matrix = 256 × 192, NEX = 1. DTI: slice thickness = 5 mm, slice gap = 0 mm, TR = 7000 ms, TE = mini, FOV = 24 cm × 24 cm, Matrix = 96 × 128, NEX = 2, Phase FOV = 1.

### MRI Analysis

**FIGURE 1:**
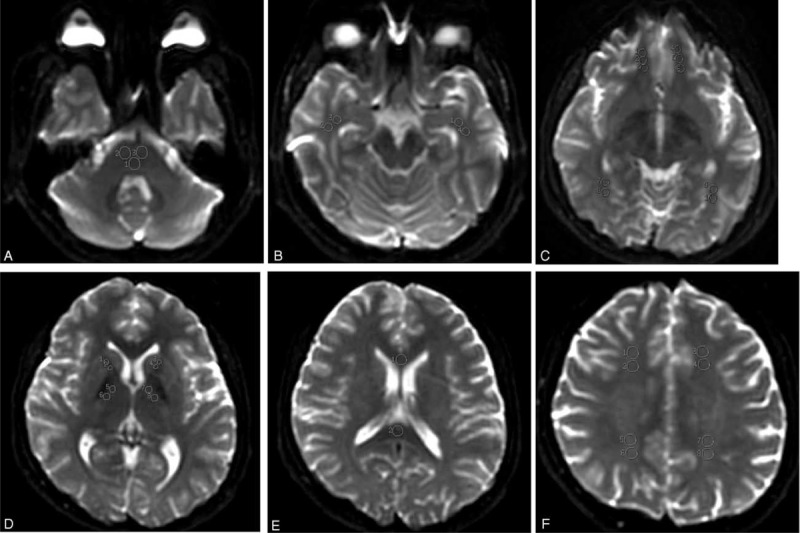
Positions of the NAWM ROIs on null images. NAWM of brainstem (A), NAWM of temporal lobe (B), NAWM of orbitofrontal and occipital lobes (C), NAWM of the anterior and the posterior limbs of the internal capsules (E), NAWM of the genu and splenium of the corpus callosum (F), NAWM of frontal and parietal lobes (F).

Conventional MRI images were first analyzed to determine the quantity, location, and size of abnormal signal intensity. DTI images were processed on an advanced workstation 4.5 with Functool 2 software. EPI distortions correction was applied before the reconstruction of the apparent diffusion coefficient (ADC) and FA maps. The ADC and FA values of NAWM were measured for all subjects, according to the method described by Lin et al.^19^ The size of ROIs ranged from 50 to 150 mm^2^, depending on the respective anatomic region. ROIs were placed on the brainstem, the genu, and the splenium of the corpus callosum, the anterior and the posterior limbs of the internal capsules, and the subcortical NAWM of the orbitofrontal, frontal, parietal, occipital, and temporal lobes. Totally 17 ROIs were measured. ROIs at brainstem were placed on the section displaying the maximum brachium pontis. ROIs of other cerebral regions were placed on 1 to 3 consecutive sections on which they were fully volumed and best displayed depending on the respective anatomic structure. The positions of the NAWM ROIs are shown in Figure 1. Three ROIs were placed on each structure. The mean value of ADC and FA was calculated as the final measurement value. To minimize the contribution of partial volume effects of CSF, vessels, and skull, every ROI was cautiously superimposed on the cerebral regions, while avoiding the abnormal regions on T2WI. All images were analyzed independently by two experienced neuroradiologists with an experience in MRI of 8 and 10 years, respectively.

### Electrophysiological Examination

Electrophysiological examinations including visual evoked potential, brainstem auditory evoked potential, somatosensory evoked potential, and blink reflex of patients and controls were measured 1 week before MRI examination.

### Statistical Analysis

All statistical analysis was performed using a commercially available SPSS software package (Version 19.0; IBM, Inc., Armonk, NY). Reliability of the measurements was first investigated by single measure intraclass correlation after the DTI parameters measured by 2 neuroradiologists independently. The desired correlation coefficient threshold was set as 0.90. Mann–Whitney *U* test was used to compare the inter-group ADC values and FA values between the patient and control groups. Receiver operating characteristic (ROC) was used to assess the ability of DTI in differentiating different groups and seeking for the optimal sensitivity and specificity. Sensitivity and specificity of conventional MRI and electrophysiological examinations was calculated using gene test as the gold standard. Bonferroni correction was used for multiple comparisons. Spearman correlation coefficient analysis was used to detect the existence of correlations between the MRI findings, patient age, and disease duration. *P* < 0.05 was considered to be statistically significant.

## RESULTS

### Conventional MRI Results

**FIGURE 2:**
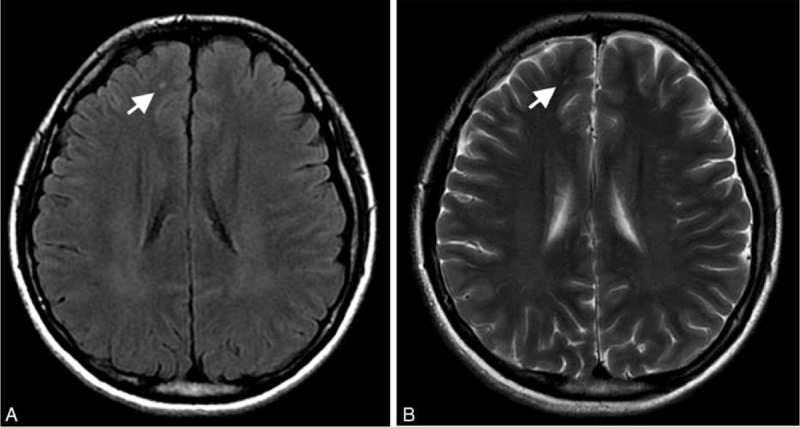
Axial T2 FLAIR (A), T2WI (B) of a 29-year-old female patient with HNPP. Hyperintense focus (white arrow) is observed in subcortical white matter of right frontal lobe on T2 FLAIR and T2WI.

Six out of 12 patients showed detectable hyperintense foci on cerebral WMs on T2WI and FLAIR images. In the 6 cases, 41 punctiform hyperintense foci were observed in bilateral frontal subcortical WM (38 foci, 92.68%), bilateral parietal WM (2 foci, 4.88%), and right temporal subcortical WM (1 foci, 2.44%) on T2WI and FLAIR. The size of hyperintense foci was 3 to 45 mm^2^. The Axial T2 FLAIR, T2WI of a 29-year-old female patient is presented in Figure 2. Hyperintense focus was observed in subcortical white matter of right frontal lobe on T2 FLAIR and T2WI. All the controls demonstrated normal appearance on conventional MRI.

### FA and ADC Values in NAWMs

**FIGURE 3:**
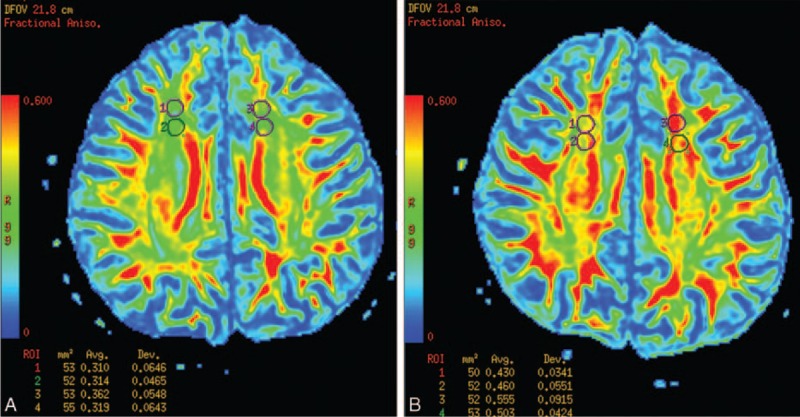
The FA maps of a patient (A) and a matching healthy control (B). The FA values of the patient in bilateral frontal NAWMs was lower compared to the control.

The reliability of ADC and FA values in all subjects ranged from 0.913 to 0.945 as established by single measure intraclass correlation. Therefore, the mean value of the measurements by 2 radiologists was considered as the final measurement value. The FA values of the patient group in bilateral frontal (L, *P* = 0.018, R, *P* = 0.006), orbitofrontal (L, *P* < 0.001, R, *P* < 0.001), and temporal (L, *P* = 0.002, R, *P* = 0.004) NAWMs significantly decreased compared with the control group. However, no statistically significant difference (*P* > 0.05) was observed in ADC values or FA values in the other regions between the patients and controls (Table 2). The FA maps of a patient and a matching healthy control are illustrated in Figure 3. Regarding to the values of controls, the normal limits (mean ± 2 SD) was set. We compared the DTI result of each patient with the normal limits and calculated the distribution of abnormal FA value. A total of 91.67% (11/12) patients showed abnormal FA values in the frontal lobe (including frontal and orbitofrontal NAWMs), with 66.67% patients (8/12) experiencing abnormal FA values in the temporal NAWMs.

**TABLE 2:**
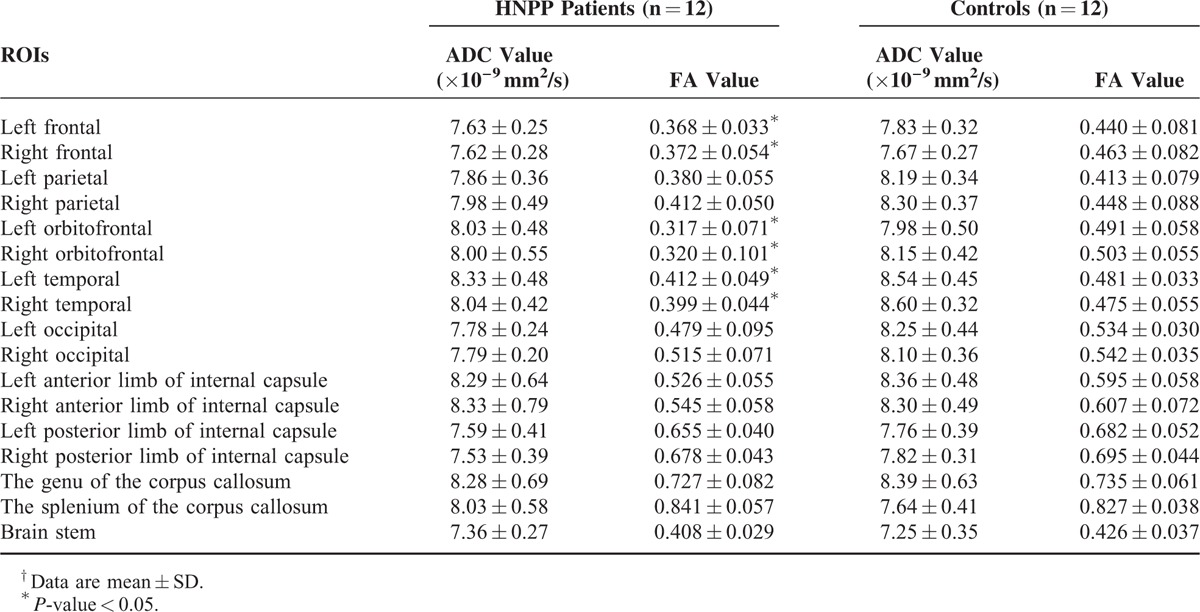
Detailed DTI Data of HNPP Patients and Controls^†^

### Electrophysiological Examination Results

All the controls demonstrated normal results. HNPP patients showed abnormal results including reduced amplitude, prolonged latency, poorly differentiated waveform, and bimodal waves. Compared to the normal values of healthy individuals (in the same age group) set up by EMG/evoked potential department of our hospital, the electrophysiological examination results of patients and controls showed no statistical significant difference (*P* > 0.05). Regarding to the values of healthy individuals, the normal limits was set. The electrophysiological examination results of each subject were compared to the normal limits, and the number of subjects with abnormal electrophysiological results was counted.

### Comparison of Sensitivity and Specificity Among Conventional MRI, DTI, and Electrophysiological Examination

**FIGURE 4:**
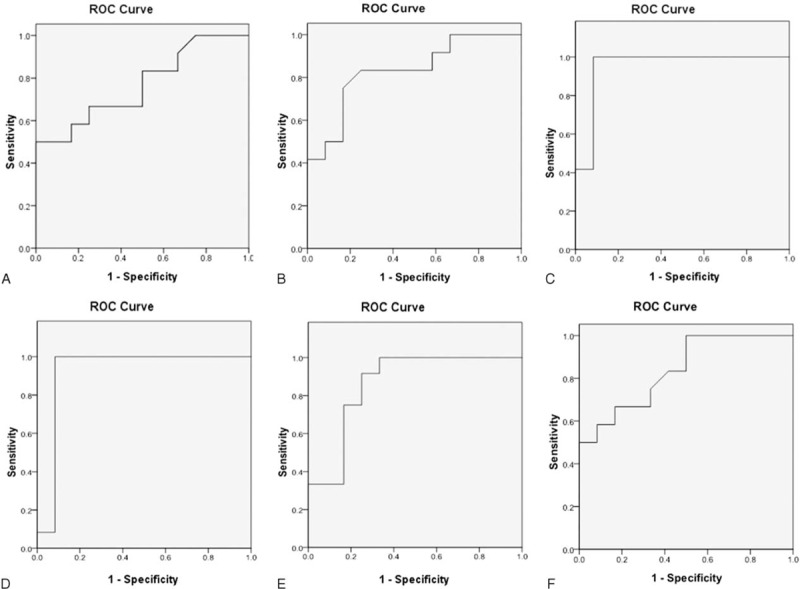
ROC curves of FA values in left frontal (A), right frontal (B), left orbitofrontal (C), right orbitofrontal (D), left temporal (E) and right temporal (F) NAWMs. AUC of FA value in left orbitofrontal NAWM is largest.

ROC analysis was performed to FA values which showed significant difference between patients and controls. Areas under ROC curve (AUC) of FA values in left frontal, right frontal, left orbitofrontal, right orbitofrontal, left temporal, and right temporal NAWMs were 0.767, 0.830, 0.951, 0.924, 0.861, and 0.837, respectively. The ROC curves are shown in Figure 4. Sensitivity and specificity of FA of left orbitofrontal NAWM were 100% and 91.7% when the cutoff value set as 0.388. The detailed data of sensitivity and specificity analysis of conventional MRI and electrophysiological examination is shown in Table 3. The specificity of conventional MRI and electrophysiological examination were all 100% due to the paired design.

**TABLE 3:**
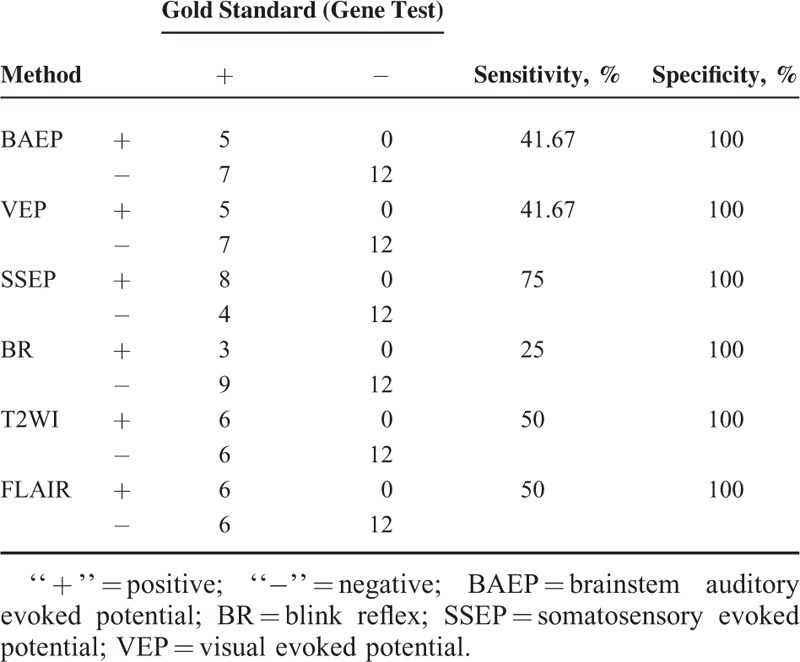
Sensitivity and Specificity Analysis of Conventional MRI and Electrophysiological Examination

### Correlations Between Conventional MRI Results, DTI Measurements, and Clinical Findings

No correlation was observed between the number of foci detected by T2WI and FLAIR imaging and DTI parameters, patient age, nor disease duration (*P* > 0.05).

## DISCUSSION

In the present study, abnormal intensity lesions on WM on T2WI and T2 FLAIR images were observed, and the decreased FA values of NAWMs in several cerebral regions in HNPP patients were observed as well. FA value is a sensitive metric to detect and quantitatively evaluate cerebral microstructural changes in HNPP. Additionally, the distribution of abnormal changes of cerebral regions in HNPP patients was described by performing conventional MRI and DTI. Most lesions were observed localized to the frontal subcortical WM.

Several previous studies have reported the presence of hyperintense foci in cerebral WM on conventional MRI.^9–12^ Twelve out of 15 reported patients were observed with hyperintensities on T2WI or FLAIR, and most of lesions were distributed in frontal and parietal regions (lesions were not described in more detail). Consistent with the previous studies, cerebral involvement was confirmed in HNPP patients by conducting MRI examination in our study. It was observed that the FA values of HNPP patients significantly decreased in several cerebral NAWM regions while compared with controls, indicating that NAWMs in HNPP patients to be microscopically abnormal. The results of our study may deepen the understanding of HNPP. DTI is capable of measuring the movement of water molecules on a microscopic scale well beyond typical MR imaging resolution, which can display subtle lesions before the detection on conventional MRI.^16^ DTI is noninvasive and provides more specific pathological markers, which reflect the state of myelination and axonal integrity. Indeed, DTI has played an important role in the assessment of WM changes in demyelination or neurodegeneration diseases such as multiple sclerosis^16–18^ and Alzheimer disease.^20,21^ Decreased FA suggests the impairment of the microstructure of brain tissues. The peripheral nerve biopsy^3^ of HNPP patients demonstrates recurrent demyelination and myelin reduplication. Horowitz and colleagues^3^ have also shown acute axonal degeneration in HNPP, consistent with the electrophysiological evidence which indicated focal axonal loss.^22,23^ In addition, teased nerve fibers of sural nerve biopsies from HNPP patients demonstrate the evidence of axonal compression and deformation.^24^ Demyelination, a lower axonal packing density, decreased axonal number, and intravoxel orientation difference caused by axonal compression and deformation will contribute to the relatively decreased FA values.^25^ The PMP22 gene deletion has been considered to be the cause of HNPP, which is also observed in CNS.^7,15^ Therefore, although HNPP is a peripheral nerve disease, it is assumed that the CNS will show similar pathological changes to peripheral nerves and these changes may induce decreased FA. It is an audacious assumption which requires verification through CNS biopsy. These microstructure impairments in NAWM cannot be detected by conventional MRI due to the demonstration of normal signal on T2WI and FLAIR images, whereas, such impairment can be detected by FA value. Consistent with Chanson et al,^15^ we also observed decreased FA in patients with PMP22 gene mutations, although the sites of involvement differed. The inconsistency in sites may be due to the small patient number and varied methods of scanning and analysis. For instance, Chanson et al^15^ obtained FA images from multiple subjects to be aligned by projection onto an alignment-invariant tract representation, while we manually delineated ROIs on NAWM. Because of the partial volume effects due to CSF, FA values from small structures were not obtained. However, due to the limitation in sample size, the generalization of the results may require further investigation. To the best knowledge, this is the first study to investigate NAWM of patients with HNPP by DTI.

Sensitivity of FA value is higher than conventional MRI and electrophysiological examination. It means that of FA value is the most sensitive metric in detecting cerebral changes associated with HNPP. The subtle structural changes in brains of patients with HNPP can be sensitively detected by FA value. Due to the paired design, the specificity of conventional MRI and electrophysiological examination were all 100%, it is meaningless to compare it among conventional MRI, DTI and electrophysiological examination. Given a specificity of as high as 91.7%, FA value has good potential in diagnosis for HNPP. From the ROC analysis, we can see that FA value of left orbitofrontal is the best index in discriminating HNPP patients with healthy people. The cutoff value for diagnosing HNPP is 0.388. FA value can quantitatively evaluate the cerebral microstructural changes in HNPP. DTI may thus be considered as a novel method for detecting cerebral impairments in HNPP patients.

Previous studies on HNPP patients performed MRI to detect cerebral abnormalities and have detected patchy hyperintense lesions in WM.^9–12^ Because of the limited cases, predilection site was not calculated in those studies. In the present study, 50% of HNPP patients showed punctiform hyperintense lesions on conventional MRI. It was observed that most lesions were located in the frontal subcortical WM on conventional MRI, paralleling the finding that frontal lobe were associated with most abnormal FA values. Hence, the frontal lobe may be the most commonly involved predilection site in HNPP. The mechanisms underlying this specific distribution remain uncertain and require further investigation.

In the present study, there was not any significant correlation between the MRI findings, patient age, or disease duration. Since cause of HNPP may be the deletion of the PMP22 gene,^7^ the resulting damage to the brain may occur at a very young age. However, our study suggests that the extent of brain damage did not increase with age. Patients in our study demonstrated CNS symptoms such as dizziness, impaired vision, seizures of hyperhidrosis. However, the pathological signs of patients were negative and MMSE scores were within the normal range, thus, the observed symptoms were considered subjective and aspecific. The electrophysiological examination results of patients did not show any statistical significance compared with normal values. Hence, there was no correlation concluded between CNS symptoms and MRI findings. In spite of this, it was assured that the WM of HNPP demonstrated abnormalities, which were detectable by performing DTI. Further studies such as pathological changes of brain in HNPP patients are required. There was no comparison between electrophysiological examinations and WM impairments because no statistical significant difference was shown on electrophysiological examination results.

There are several limitations in the present study. Firstly, due to the rareness of HNPP, the sample size was limited and hence may affect the statistical power. The generalization of the derived results may require a larger group study in the future. Secondly, due to the lack of brain biopsies of HNPP patients, there was not a pathology-imaging comparison made. Therefore, the WM changes on pathological level remained unclear. Thirdly, there may be a bias in the patient group that only patients with symptoms were enrolled in this study, whereas patients in the early stage of the disease without clear symptoms were not included, due to limited access to the patient population. Lastly, the dynamic changes in the brains of HNPP patients were not studied, which would be interesting extension of the work in the future.

## CONCLUSIONS

This study confirmed cerebral involvement in HNPP patients. The impairment of the microstructure of brain tissue was found by DTI. FA value is a sensitive metric to detect and quantitatively evaluate cerebral microstructural changes in HNPP. The best biomarker is FA value of left orbitofrontal WM and the cutoff value for diagnosing HNPP is 0.388. In addition, the frontal lobe is the predilection site that is most involved in HNPP.
